# One-step loop-mediated isothermal amplification (LAMP) for the rapid and sensitive detection of *Fusarium fujikuroi* in bakanae disease through *NRPS31*, an important gene in the gibberellic acid bio-synthesis

**DOI:** 10.1038/s41598-019-39874-z

**Published:** 2019-03-06

**Authors:** S. Y. Zhang, D. J. Dai, H. D. Wang, C. Q. Zhang

**Affiliations:** 1 Department of Plant Pathology, Zhejiang Agriculture and Forest University, Hangzhou, 311300 China; 2 Institute for the Control of Agrochemicals of Zhejiang Province, Hangzhou, 310020 China

## Abstract

Rice bakanae disease caused by *Fusarium fujikuroi* is one of the most famous seed borne diseases. If infected seeds are used, this disease will occur with serious impacts. Thus, a simple, reliable, specific and sensitive method for surveillance is urgently needed to screen infected seeds and seedlings at early developmental stages. In this study, a rapid and efficient loop-mediated isothermal amplification (LAMP) method was developed to detect *F*. *fujikuroi* in contaminated rice seeds and seedlings for diagnosis of bakanae disease. *NRPS31* gene plays an important role in the gibberellic acid (GA) bio-synthesis of *F*. *fujikuroi*, and is not present in any other sequenced fungal genome, and thus was adopted as the target for LAMP primer design. The LAMP assay enables the fast detection of as little as 1 fg of pure genomic *F*. *fujikuroi* DNA within 60 minutes. Further tests indicated that the LAMP assay was more sensitive and faster than the traditional isolation method for *F*. *fujikuroi* detection in rice seeds and seedlings. Our results show that this LAMP assay is a useful and convenient tool for detecting *F*. *fujikuroi*, and it can be applied widely in seed quarantine of bakanae disease, providing valid data for disease prevention.

## Introduction

Rice (*Oryza sativa* L.) is an essential staple food consumed worldwide. A recent survey by the International Food Policy Research Institute indicates that rice production will need to increase 38% by 2030 to feed the expanding human population but available arable land is being lost to housing and industrialization. Rice bakanae disease (RBD) caused by seed-borne *Fusarium fujikuroi* results in serious economic losses in rice growing countries^1–6^. RBD is one of the most serious and oldest problems in rice productions, and was first described in 1828 in Japan^7^. RBD leads to a significant production loss of up to even 50% of rice yields. In 2011, up to 40% disease incidence was reported from the Kapurthala, Ropar, Patiala, Ludhiana, Amritsar, Gurudaspur and Hoshiarpur districts of Punjab, India^8^. In Korea, 2.9% of the rice seedlings in seed boxes were infected by RBD in 2003, and a major increase to 28.8% was documented in 2006^4^. Thus, if food security for this important crop is to be preserved, monitoring methods for *F*. *fujikuroi* in seeds are urgently needed to prevent the occurrence and spread of RBD.

RBD can affect rice from the pre-emergence stage to the mature stage, and cause elongation and upward root growth of rice plants mainly due to gibberellic acids (GAs), a family of plant hormones, which were secreted by *F*. *fujikuroi*^9–13^. RBD is transmitted mainly by seed contamination and the pathogen can survive in seeds and infected rice straw. Importantly, infested seeds will pass the pathogen to healthy seeds when they are stored together. During the disease’s cycle in rice fields, infection can occur by sowing infected seeds with non-infested seeds. As generally, seeds contaminated with the fungus provide the initial source for secondary infection. Under favorable environmental conditions, infected plants have the capacity to produce numerous conidia that subsequently infect healthy plants, resulting in major yield loss^14,15^. Thus, early detection of *F*. *fujikuroi* in seeds and seedlings is essential to prevent the occurrence and spread of RDB. Several methods for surveillance of RBD, including *F*. *fujikuroi* isolation, seed morphology scanning, and polymerase chain reaction (PCR) detection, are among the common practical methods adopted for RDB diagnosis in the laboratory^8,16^. However, these traditional methods are unsuitable for field applications, as they require technical expertise, specialized equipment and can be time-consuming. Technologically, the extraction and consequent molecular detection of genomic DNA from *F*. *fujikuroi* contaminated seed samples is usually difficult to be exerted. It can be ascribed to the complicated biochemical components existed in seeds, including not only the genomic DNA, but also some microbes and overwhelming number of PCR inhibitors. Taking such disadvantages into account, a DNA amplification technique known as loop-mediated isothermal amplification (LAMP) has been developed in this study for the detection of *F*. *fujikuroi*.

LAMP was invented and applied as early as in 2000 and is recognized as a user-friendly, rapid, and efficient amplification method of DNA sequences at a single temperature, that is both sensitive and specific^17^. This technique is less sensitive to inhibitors than PCR and, hence, has been applied for detection of several plant-pathogens, including *Didymella bryoniae* from cucurbit seeds^18^ and *Colletotrichum truncatum* from soybeans^19^. The LAMP assay employs four to six oligonucleotide primers and the strand displacement activity of *Bst* DNA polymerase to amplify specific DNA sequences with high specificity^20^. The large quantity of amplified product and by-product (magnesium pyrophosphate) obtained via the LAMP reaction allows effective detection of target DNA based on visual assessment of turbidity, or a color change that develops upon addition of color-changing reagents^21^. LAMP products can also be visualized as banding pattern on agarose gel^22^. Overall, without any special equipment, LAMP assays can amplify DNA with high specificity and efficiency compared with conventional PCR. In this study, we developed a specific and efficient method for one-step detection method of *F*. *fujikuroi* in rice seeds based on the non-ribosomal peptide synthetase (*NRPS31*),which is conserved and unique to *F*. *fujikuroi* and plays an important role in the GA bio-synthesis.

## Results

### LAMP primers

**Figure 1:**
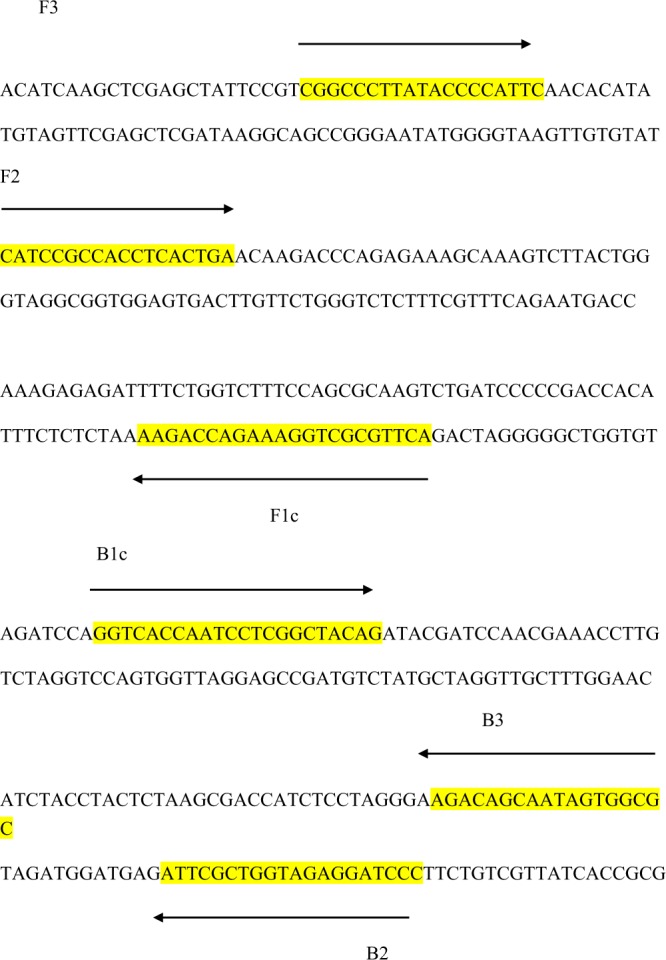
Partial sequence of non-ribosomal peptide synthetase (*NRPS31*) of *Fusarium fujikuroi* and the location of the Loop-Mediated Isothermal Amplification (LAMP) primers of Fns31–1. Arrows indicate the direction of extension. By targeting six conserved regions of *NRPS31* (F3c, F2c, Flc, B1, B2, B3), four specific primers,including two outer (F3 and B3) and two inner, FIP (Forward Inner Primer, F1c and F2) and BIP (Backward Inner Primer, B1c and B2) primers were designed. F1c is the complementary sequence of F1.

The LAMP primers (Fig. 1, Table 1) for *F*. *fujikuroi* were checked by comparison with all available relevant sequences. The primers were chosen to allow specific amplification of *F*. *fujikuroi* and did not show any similarities to other sequences available in NCBI GenBank database. During the design of LAMP primers, ΔG values of the 3′ ends F3/B3 primer and F2/B2 primer, 5′ ends of the F1c and B1c primer were determined and the values were −4.51, −4.74, −4.91, −5.26, −5.40 and −5.51 Kcal/mole, and all ΔG values were less than −4 Kcal/mol. Finally, a set of four primers exhibiting high species specificity and sensitivity which targeted the *NRPS31* sequence of *F*. *fujikuroi* were selected for further study. The selected target for the LAMP assay was located on non-ribosomal peptide synthetase (HF679023.1, position 6544644 to 6544870 bp).

**Table 1:** Primers Fns31-1 used for development of a loop-mediated isothermal amplification (LAMP) assay for specific detection of *Fusarium fujikuroi*.

Primer type	Sequence (5′-3′)
F3	CGGCCCTTATACCCCATTC
B3	GCGCCACTATTGCTGTCT
FIP (F1c-F2)*	ACTTGCGCTGGAAAGACCAGAACATCCGCCACCTCACTGA
BIP (B1c-B2)	GGTCACCAATCCTCGGCTACAGCCCTAGGAGATGGTCGCTTA

*FIP is a hybrid primer consisting of the F1c sequence and the F2 sequence, BIP is a hybrid primer consisting of the B1c sequence and the B2 sequence.

### Specificity and sensitivity of the LAMP assay

**Figure 2:**
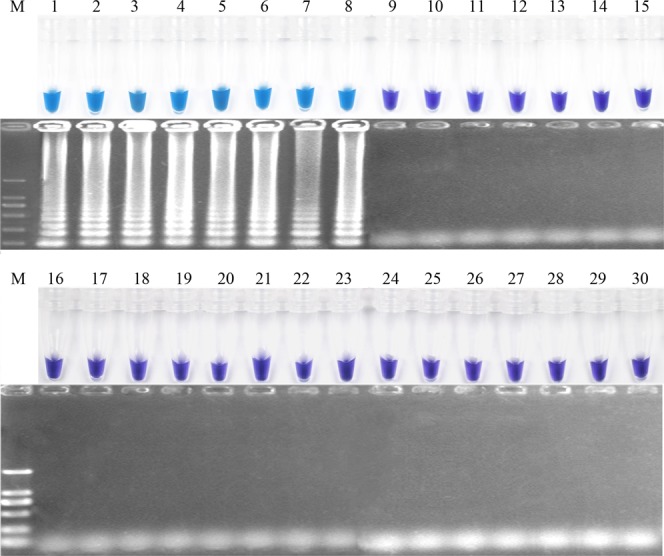
The specific loop-mediated isothermal amplification of *Fusarium fujikuroi* by the primers Fns31–1. (**A**) Assessment based on HNB visualization of color change of the LAMP products; (**B**) Agarose gel electrophoresis of LAMP products. M, DNA marker; 1. *F*. *fujikuroi*; 2. *F*. *fujikuroi*; 3. *F*. *fujikuroi*; 4. *F*. *fujikuroi*; 5. *F*. *fujikuroi*; 6. *F*. *fujikuroi*; 7. *F*. *fujikuroi*; 8. *F*. *fujikuroi*; 9. *F*. *avenaceum;* 10. *F*. *semitectum*; 11. *F*. *verticillioide*; 12. *F*. *lateritium*; 13. *F*. *sambucinum*; 14. *F*. *culmorum*; 15. *F*. *sporotrichioides*; 16. *F*. *oxysporum*; 17. *F*. *proliferatum*; 18. *F*. *solani*; 19. *F*. *graminearum*; 20. *Curvularia lunata*; 21. *Aspergillu terreus*; 22. *Sclerotinia sclerotiorum*; 23. *Bipolaris sorokiniana*; 24. *Phomopsis asparagi*; 25. *Penicillium* sp.; 26. *Ustilaginoidea virens*; 27. *Pyricularia oryzae*; 28. *Alternaria alternata*; 29. *Rhizoctonia solani*; 30. nuclease-free water.

The specificity of the primers was tested with *F*. *fujikuroi* isolates and no-target DNA samples of different pathogenic and nonpathogenic fungi.With the addition of 0.15 μM Hydroxynaphthol blue (HNB), the results of LAMP assay can be visualized via color shift from violet to blue. The *F*. *fujikuroi* isolates tested positive in every replicated test, indicated by color changes from violet to azure evidently of the reaction solution, whereas the original violet color was retained for other fungi (Fig. 2A). The nuclease-free water templates showed no color change in any validation test. Moreover, a ladder-like pattern in gel electrophoresis of the LAMP amplified products revealed similar findings to the color change (Fig. 2B). Consequently, the newly developed LAMP assay employing the primer Fns31-1 (Table 1) showed high specificity in detection of *F*. *fujikuroi*.

**Figure 3:**
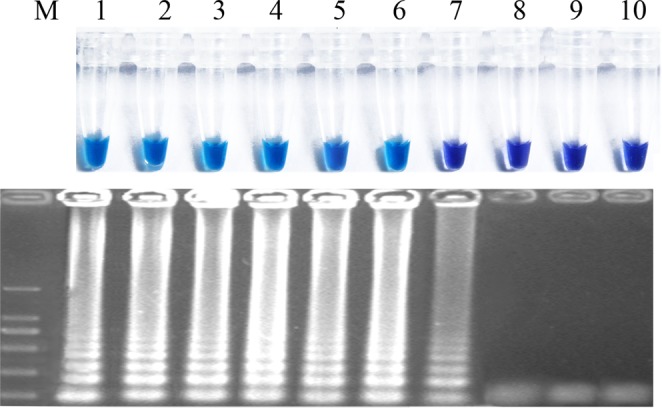
The results of loop-mediated isothermal amplification (LAMP) with different concentration of DNA template. (**A**) Assessment based on HNB visualization of color change of the LAMP products. (**B**) Assessment based gel electrophoresis analysis of the LAMP products. M, DNA marker; 1. 1 ng/μl; 2, 100 pg/μl; 3, 10 pg/μl; 4, 1 pg/μl; 5, 100 fg/μl; 6, 10 fg/μl; 7, 1 fg/μl; 8, 100 ag/μl; 9, 10 ag/μl; 10, nuclease-free water.

After it was determined that the primer Fns31-1 was specific for *F*. *fujikuroi*, the lowest detection limit was characterized using 10-fold serial dilutions of pure *F*. *fujikuroi* DNA (1 ng to 10 ag) extracted from three separate isolates *F*. *fujikuroi*. The lowest detection limit for *F*. *fujikuroi* was per reaction within 60 min incubation time when using template DNA extracted from pure cultures10 fg and 1 fg, respectively for color shift through addition of HNB and gel electrophoresis (Fig. 3). As a comparison, conventional PCR conducted with primers Fns31-1-F3/Fns31-1-B3 exhibited 100 times higher than LAMP. The results indicated that, compared to the PCR method, our LAMP assay was more sensitive.

### LAMP detection of *F*. *fujikuroi* in rice seeds

The efficiency of the LAMP assay in detecting *F*. *fujikuroi* in rice seeds was tested by inoculating pathogen-free samples of rice seeds with *F*. *fujikuroi*. The results of LAMP assay were positive with inoculated treatments at a mixing ratio (inoculated rice seeds:non-inoculated healthy seeds) of 1:199, while none of the non-inoculated treatments showed any reaction. This was supported by similar results of conventional methods of fungus isolation. Typical fungal colonies distributed around each seed in the plates. As the mixing ratio decreased to 1:399, the LAMP assay was shown to provide substantially more positive results than those of fungus isolation. At the ratio of 1:3199, for example, the detection rate (%) for fungus isolation was only 0.67 but that of LAMP was as high as 16.67 (Table 2). This result indicated that the LAMP assay was more sensitive than the traditional isolation method.

**Table 2:** The detection rate of LAMP assay and conventional microbiological isolation method for *Fusarium fujikuroi* at different levels of infestation.

Detection method	Detection rate (%)^b^ at different mixing ratio^a^					
	1:99	1:199	1:399	1:799	1:1599	1:3199
isolation	100.00*^c^	100.00*	77.00*	12.00*	0.67*	0.67*
LMAP	100.00*	100.00*	96.67**	90.00**	46.67**	16.67*

^a^Mixing ratio means mixing the number of inoculated rice seeds: the number ofnon-inoculated healthy seeds. For example, 1:99 means one inoculated rice seed was mixed with 99 non-inoculated healthy seeds.

^b^For each mixing ratio, 20 seeds were used at random to detect the *Fusarium fujikuroi* for each seed. Detection rate (%) means numbers of seeds for which F. fujikuroi was detected out of 20 seeds.

^c^Mean values with the different number of asterisks within the same column were significantly different (*t* tests, *P* < 0.01).

### LAMP detection of *F*. *fujikuroi* in rice seedlings

**Figure 4:**
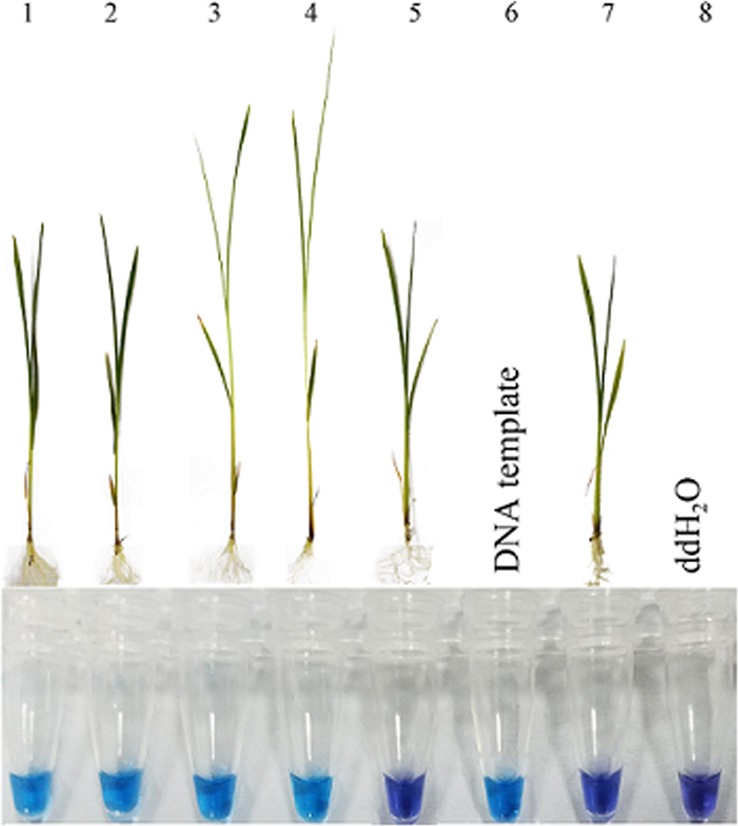
The detection of inoculated seedlings by loop-mediated isothermal amplification. 1–4, DNA from seedlings samples incubated with 1 × 10^3^, 1 × 10^4^, 1 × 10^5^ and 1 × 10^6^ conidia ml^−1^; 5, DNA from inoculated seedlings samples treated with 3 μg/ml of phenamacri; 6, the DNA template of *Fusarium fujikuroi*; 7, DNA from non-infested seedlings as a negative control; 8, negative control (no seedling).

After elongation symptoms were observed in the seedlings incubated with inoculum suspension, the seedlings treated with phenamacril and negative control were still healthy. The results of LAMP assays from seedlings incubated with inoculum suspension of *F*. *fujikuroi* at the concentration from 1 × 10^3^ conidia ml^−1^ to 1 × 10^6^ conidia ml^−1^ were all positive.The seedlings inoculated with 1 × 10^6^ conidial ml^−1^ suspension and treated with 3 μg/ml of phenamacril were all negative (Fig. 4). When compared to traditional isolation and culture methods, the LAMP assay was more accurate and showed higher sensitivity. Among the inoculated samples with different concentration of conidia, *F*. *fujikuroi* was only re-isolated from seedlings inoculated with 1 × 10^5^ and 1 × 10^6^ conidia ml^−1^ with the respective frequency of 63.2% and 87.6% but not isolated from inoculations with 1 × 10^3^, 1 × 10^4^ conidia ml^−1^.

**Figure 5:**
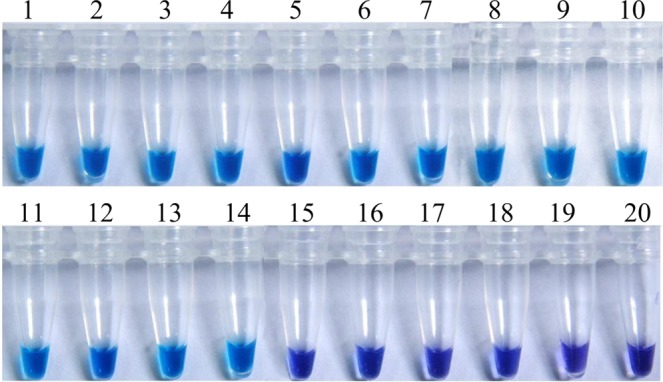
The detection from stems infected rice bakanae disease collected from the field by loop-mediated isothermal amplification. 1–13, samples of the bakanae disease in the field; 14, the DNA template of *Fusarium fujikuroi*; 15–19, healthy plant; 20, nuclease-free water.

As shown in Fig. 5, the color of the naturally diseased seedling and positive control sample was sky blue and the color of the negative control and healthy samples was violet, however, amplification was never observed in healthy seedling and negative positive control samples. For each of the two sites, Shaoxing and Jinhua,13 infected seedlings randomly chosen all showed positive. For the traditional isolation method, *F*. *fujikuroi* was successfully isolated from 7 infected seedlings with the frequency of 53.8%. The results show that, compared to the traditional isolation method, our LAMP assay was more rapid and sensitive for field samples.

## Discussion

RBD caused by *F*. *fujikuroi* is an important seed-borne disease that is common in the primary rice production regions. Traditional isolation and culture methods are important in the diagnosis of plant fungal diseases. However, because of morphological similarities, it is difficult to distinguish *F*. *fujikuroi* from other *Fusarium* species by microscopic observation alone, which can be lengthy and require special training. Meanwhile, our research shows it is hard to efficiently and accurately assay seed samples if the infection rate by *F*. *fujikuroi* is below 0.25%. The seedling blotter assay was widely employed in seed health tests for *Fusarium* sp., however, it requires large seed sample sizes to ensure reliability of the test result. Until present, the recommended method by ISTA (the International Rules for Seed Testing) (http://www.bibme.org/citation-guide/apa/web-site/) requires blotting a sample of 400 rice seeds,evenly dividing onto 16 filter paper (90-mm) soaked with distilled water. After incubation at 22 °C in 12 h cycles of light and darkness for 7 d, each seed was examined and confirmed by stereoscopic and the percentage of infected seeds was recorded. Hence, visual, rapid and accurate seed health testing technique could contribute meaningfully to eliminate infested seed lots and thereby minimize the threat of outbreaks of this disruptive disease.

The several existing assays developed to test seeds for *F*. *fujikuroi* require long incubation periods (blotter assay), or expensive equipment (PCR and real-time PCR). In contrast to traditional methods of pathogen detection in plant tissues, the LAMP assay is simple and requires no special techniques, specialized equipment and knowledge. Only the primers, reagents, and a temperature-controlled device are needed to perform the LAMP reactions. When the LAMP products were detected through gel electrophoresis,a ladder-like pattern of the amplified products was observed in all assays. Moreover, the results can be easily visualized with the addition of HNB in LAMP assay, which enables a clear and easy detection of positive samples by unaided eye via color shift from violet to blue. Furthermore, this modification did not diminish either sensitivity or specificity of the reaction^23^.

Previous reported LAMP assays for fungi mostly target regions of high similarity among species such as ITS^24,25^ which tend to have less interspecific variability and possibly hamper the development of species-specific primers. Our LAMP assay for the detection of *F*. *fujikuroi* employed four specific LAMP primers, which were designed, based on the sequence of the conserved *NRPS31*gene. This gene is conserved and unique to *F*. *fujikuroi* and plays an important role in the GA bio-synthesis^26^. Thus, *NRPS31* gene was a highly specific target for the design of LAMP primers for the detection of *F*. *fujikuroi*. Generally, the seeds may contain many species of *Fusarium*, *Penicillium*, *Rhizopus*, *Alternaria* and *Ustilaginoidea*, however, the results provided in this study clearly indicated that the LAMP method diminished the possibility of cross-reactivity due to the specificity of the primers.

The sensitivity of the LAMP assay with genomic DNA of *F*. *fujikuroi* was 10 fg and 1fg respectively for color shift through addition of HNB and gel electrophoresis, and the sensitivity was further verified on plant samples. The reported TaqMan real-time PCR and SYBR Green real-time PCR assay had the respective detection limit of 27.5 fg and 10 pg of *F*. *fujikuroi* DNA^27,28^. Upon detecting for seed and seedling samples, the LAMP assay yielded satisfactory results compared to traditional isolation and culture methods. This rapid, simple and cost-effective LAMP assay also overcomes limitations frequently encountered when using PCR assays for detection of *F*. *fujikuroi* and other slow-growing fungal pathogens in seeds. In summary, the LAMP method targeting *NRPS31* gene which was conserved and unique to *F*. *fujikuroi* was successfully developed for detection of *F*. *fujikuroi* in pure culture, rice seedlings and seeds, showing excellent sensitivity, specificity, simplicity and user-friendly handling compared with conventional methods.

## Materials and Methods

### Fungal isolates, culture conditions and DNA extraction

*F*. *fujikuroi* isolates were isolated from diseased rice seedlings in Shao xing (120°65s, 29°98w), and Jia xing (120°86s, 30°75w) Zhejiang Province, China, and the isolates were identified using both morphological^16^ and molecular approaches, using sequencing translation elongation factor 1-α^29,30^ and maintained in potato dextrose agar (PDA) slants in dark. Other twenty-one rice seed-associated fungi^31,32^ were bought from Agricultural Culture Collection of China (ACCC), China Center of Industrial Culture Collection (CICC) or China General Microbiological Culture Collection Center (CGMCC) and accession codes and host type are listed in Table 3. These strains were adopted to provide comparison to confirm the specificity of proposed methods for *F*. *fujikuroi*. Prior to experiments, all isolates were transferred to PDA plates and were incubated for 5 d at 25 °C in darkness. Genomic DNA was extracted from each sample using a Rapid Fungi Genomic DNA Isolation Kit (Sangon, Shanghai, China) according to the manufacturer’s instructions. The quality of the DNA was checked in agarose gels (1.7%) and the quantity determined in a spectrophotometer (NanoDrop Technologies, Wilmington, Delaware, USA).

**Table 3:** Fusarium and other fungal isolates used to evaluate the analytical specificity of the LAMP assay for detection of *Fusarium fujikuroi*^a^.

Species	Isolate	Host	Location
*Fusarium fujikuroi*	FFSX-05	Rice	Zhejiang, China
*F*. *fujikuroi*	FFSX-20	Rice	Zhejiang, China
*F*. *fujikuroi*	FFJX-14	Rice	Zhejiang, China
*F*. *fujikuroi*	FFJH-09	Rice	Zhejiang, China
*F*. *fujikuroi*	FFJS-16	Rice	Jiangsu, China
*F*. *fujikuroi*	FFJX-20	Rice	Jiangsu, China
*F*. *fujikuroi*	FFJX-22	Rice	Jiangsu, China
*F*. *fujikuroi*	CGMCC 3.1108^b^	Rice	Zhejiang, China
*F*. *avenaceum*	ACCC 30065^c^	Soil	China
*F*. *semitectum*	ACCC 31945^d^	Soil	Beijing, China
*F*. *verticillioide*	ACCC 37123	Rice	Gansu, China
*F*. *lateritium*	ACCC 30023	Soil	Guangdong, China
*F*. *sambucinum*	ACCC 30078	/	Beijing, China
*F*. *culmorum*	ACCC 37130	Grass seed	Gansu, China
*F*. *sporotrichioides*	ACCC 37402	Garlic	Henan, China
*F*. *oxysporum*	ACCC 30927	Rice	Hainan, China
*F*. *proliferatum*	CICC 2489^c^	Rice	Anhui, China
*F*. *solani*	ACCC 37119	Rice	Hebei, China
*F*. *graminearum*	ACCC 37680	Wheat	Jiangxi, China
*Curvularia lunata*	ACCC 36693	Rice	Anhui, China
*Aspergillu terreus*	ACCC 31880	Soil	Xinjiang, China
*Sclerotinia sclerotiorum*	ACCC 36462	Rape	Shandong, China
*Bipolaris sorokiniana*	ACCC 36805	Wheat	Beijing, China
*Phomopsis asparagi*	CICC 2706^d^	Reed	Hebei, China
*Penicillium* sp.	ACCC 31507	Soil	Shandong, China
*Ustilaginoidea virens*	ACCC 2711	Rice	Hunan, China
*Pyricularia oryzae*	ACCC 37631	Rice	Fujian, China
*Alternaria alternata*	ACCC 36843	Rice	Hainan, China
*Rhizoctonia solani*	ACCC 36246	Rice	Beijing, China

^a^*F*. *fujikuroi* isolates were positive with LAMP primer Fns31-1.

^b^CGMCC, China General Microbiological Culture Collection Center.

^c^ACCC, Agricultural Culture Collection of China.

^d^CICC, China Center of Industrial Culture Collection.

### Inoculation of rice seeds with *F*. *fujikuroi*

Seeds (cultivar XS11) were artificially infected with *F*. *fujikuroi* by a method with small modifications^18,30^. Briefly, agar plugs (0.5 cm in diameter) of *F*. *fujikuroi* (CGMCC 3.1108) were cut at the leading edge of colony growth after 7 d at 25 °C on PDA plates under continuous darkness, and three agar plugs were incubated into separate 250-ml Erlenmeyer flasks containing 20 g autoclaved rice seeds and 100 ml nuclease-free water. Subsequently, the mixtures of fungal plugs and rice seeds were co-incubated at 25 °C for 72 h at 150 rpm with alternating light and dark. Seeds exposed to the fungus were then removed from the Erlenmeyer flasks, and air-dried on sterile absorbent paper at 25 °C for 48 h.

### Generation of rice seedlings infected by *F*. *fujikuroi*

With respect to inoculation of seedlings, seed lots of rice, cultivar XS11, found free of seed-borne *F*. *fujikuroi* were used in the experiments, which were surface sterilized by method of Kim^33^ with modifications. Seeds were immersed in 4% sodium hypochlorite for 3 min and rinsed three times consecutively in sterile distilled water, and transferred to seedling tray (26 cm × 13 cm × 6 cm) containing sterile distilled water, and incubated at 57 °C for 13 min. *F*. *fujikuroi* isolate (CGMCC 3.1108) was cultured on PDA plates and incubated at 25 °C under continuous light for 7 d. We then added 5 ml of distilled water to each plate, dislodged the conidia with a cotton swab, and filtered the suspension through double-layered cheesecloth. Conidia of *F*. *fujikuroi* were collected from 7-d-old cultures on PDA and suspended in sterile distilled water. The conidial suspensions were determined using a hemocytometer and adjusted to concentrations of 1 × 10^3^, 1 × 10^4^, 1 × 10^5^ and 1 × 10^6^ conidial ml^−1^.Thirty germinated sprouts with germ length up to half of length of the seed were soaked in each concentration of the conidial suspensions for 12 h at 28 °C, 70 rpm in the dark. Negative controls were double-distilled water (ddH_2_O) in place of spore suspension. To ensure the accuracy of the results and avoid false positives, 30 sprouts inoculated with 1 × 10^6^ conidial ml^−1^ suspension, and treated with 3 μg/ml of phenamacril^34^, the most effective fungicide preventing RBD available at present, as another negative control. After inoculation, 30 sprouts were sown in nutrient solution in a growth chamber with a 12-h photoperiod and a daytime temperature of 28 °C and 25 °C at night (70–80% RH) until disease symptoms were observed. After 15 d, when the elongation and upward growth of roots were observed as seedling symptoms, the internodes of seedling stem bases were cut into small segments (length at 0.5 cm).

### Isolation of *F*. *fujikuroi* from seeds and seedlings

To assess the detection result by LAMP, traditionally isolation and culture method was used to isolate *F*. *fujikuroi* from rice seeds and seedlings. The internodes of seedling stem bases were cut into small segments (length at 0.5 cm), and immersed in sterile water to remove dirt from the surface. The segments or seeds were immersed in 4% sodium hypochlorite for 4 min and 70% alcohol for 10s successively and rinsed three times in sterile water. Finally, the samples were dried with sterile absorbent paper, and inoculated on PDA in a Petri-dish under 25 °C in the dark. The percentage of *F*. *fujikuroi*-infested was observed after 3 d (n = 200 seeds or segments)

### LAMP primers design and screen

The *NRPS31* gene is not present in any known sequenced fungal genome other than *F*. *fujikuroi* and this gene plays an important role in the GA bio-synthesis, which is necessary for pathogenesis^26^. Thus, this conserved and unique *NRPS31* gene was a highly specific target for the design of LAMP primers for the detection of *F*. *fujikuroi*. A set of LAMP primers, comprising two outer (F3 and B3) and two inner (FIP and BIP) primers were designed using the Primer Explorer V4 software program (http://primerexplorer.jp), based on the *F*. *fujikuroi NRPS31* sequence (HF679023.1). Best primer selection was based ΔG values of less than or equal to −4 Kcal/mol at the 3′ end of F3/B3 and F2/B2, and 5′ ends of F1c and B1c, and were synthesized by Sangon.

### LAMP reaction mixtures and conditions

LAMP reactions were performed using the above described primer sets shown in Fig. 1 and Table 1. Each reaction contained 0.8 μM of the primers FIP and BIP, 0.1 μM of the primers F3 and B3, 0.8 M betaine, 1.4 mM dNTPs, 20 mM TrisHCl (pH 8.8), 10 mM KCl, 10 mM (NH4)_2_SO_4_, 6 mM MgSO_4_, 0.1% (v/v) Triton X-100, 8 U of *Bst* DNA polymerase, 150 μM HNB and 1 μl of the target DNA sample extracted as described. The reaction mixtures were incubated in a heated block at an optimal temperature of 64 °C with an amplification period of 60 min followed by incubation at 80 °C for 10 min to terminate the reactions. The reaction results were examined via visual color changes of HNB (from violet to sky blue) after the reaction and/or further confirmed via 1% agarose gel electrophoresis.

### Assessment of specificity and sensitivity of LAMP assay

The specificity was determined by the LAMP assay with DNA extracted from eight *F*. *fujikuroi* and other twenty-one rice seed-borne or soil-born fungi^29^, as discussed above and listed in Table 2. To determine the sensitivity of the LAMP assay, genomic DNA from *F*. *fujikuroi* (CGMCC 3.1108) was used. The LAMP assay detection limit is defined here as the smallest amount of DNA detected in each test replicate. The LAMP assay was tested using ten-fold serial dilutions of pure isolate genomic DNA ranging from 10 ng/μl to 100 fg/μl. Dilution series were prepared in sterile deionized water. The associated LAMP assays were performed using the same conditions mentioned above. In order to obtain consistent results, each LAMP reaction was repeated in triplicate. Negative controls contained nuclease-free water in place of template DNA. All reactions were performed three times.

### DNA purification and LAMP detection from rice seeds and seedlings

To determine how effective LAMP detection was for identifying the presence of *F*. *fujikuroi*, the detection assays were conducted on infected rice seeds and seedlings. Individual *F*. *fujikuroi*-infected seed was mixed with 99, 199, 399, 799, 1599 and 3199 healthy seeds in the Erlenmeyer flasks, respectively, and incubated at 25 °C, 300 rpm for 2 h. Single healthy seed mixed with 99 healthy seeds samples served as a negative control. To evaluate the LAMP detection of *F*. *fujikuroi* from artificially infested seeds, 20 seeds were collected at random from each Erlenmeyer flask and each seed was transferred to a 1.5-ml self-standing screw-cap tube (Bio Basic Canada Inc) for the DNA extraction. DNA from each seed was extracted using a Chelex-100 protocol^35^ with modifications made as follows:200 μl of 5% Chelex-100 sodium form (Sigma-Aldrich) solution was added to each seed, which was then crushed using a sterile micro pestle. After centrifugation at 2000 rpm for 1 min, the tubes were treated for 6 s at 40 KHz in an ultrasonic bath (Desen DSA50-GL2, Fuzhou, Fujian, China), and then submerged in water bath (Sen Xin DKB-501A, Shanghai, China) at 100 °C for 5 min, with both steps then repeated again. Suspensions in the tubes were then allowed to cool to room temperature before the tubes were stored at −20 °C if not immediately used as DNA template for LAMP reactions. DNA extractions for inoculated seedlings were from small segments using the method as described above for seed samples, and stored at −20 °C until tested using the LAMP assays. All assays were done in triplicate in order to obtain consistent results. Purified DNA from *F*. *fujikuroi* on PDA was used as a positive control while DNA from non-inoculated healthy seedling was used as a negative control.

### LAMP detection of *F*. *fujikuroi* from seedlings collected in rice fields

To further confirm the efficiency of LAMP assays for the detection of *F*. *fujikuroi* from seedlings, naturally infected seedlings and healthy seedlings were collected from the fields in Shaoxing and Jinhua of Zhejiang Province. For each site, a total of 35 seedlings just occurring of symptom were sampled from different rice fields and fields were separated, at least, 30 km from each other. Five to 10 seedlings were collect from each field. These seedlings samples were brought back to laboratory for testings. For each site, 13 out of 35 seedlings was chosen at random and small segments from stems of each seedling were adopted for LAMP assay and traditional isolation of *F*. *fujikuroi* as described above.
